# PEDOT percolation networks for reversible chemiresistive sensing of NO_2_

**DOI:** 10.1039/d1ra03648c

**Published:** 2021-06-28

**Authors:** Merel J. Lefferts, Ben I. Armitage, Krishnan Murugappan, Martin R. Castell

**Affiliations:** Department of Materials, University of Oxford Parks Road Oxford OX1 3PH UK merel.lefferts@materials.ox.ac.uk

## Abstract

Detection of NO_2_ plays an important role in various safety applications. However, sensitive and reversible sensing of NO_2_ remains a challenge. Here we demonstrate the use of poly(3,4-ethylenedioxythiophene) (PEDOT) conducting polymer percolation networks for chemiresistive sensing of NO_2_. By adjusting the electrochemical polymerisation and doping conditions of the polymer, we show control over the relative contributions of oxidised and over-oxidised PEDOT to the sensing behaviour. Reversible NO_2_ sensors using only PEDOT as the sensor material are demonstrated. By operating the sensor near the electrical percolation threshold, a higher sensitivity is achieved compared to more traditional thin film based chemiresistive sensors. A limit of detection of 907 ± 102 ppb was achieved.

## Abstract 2

Reversible NO_2_ sensing by controlling the balance of oxidised and over-oxidised PEDOT.

## Graphical abstract

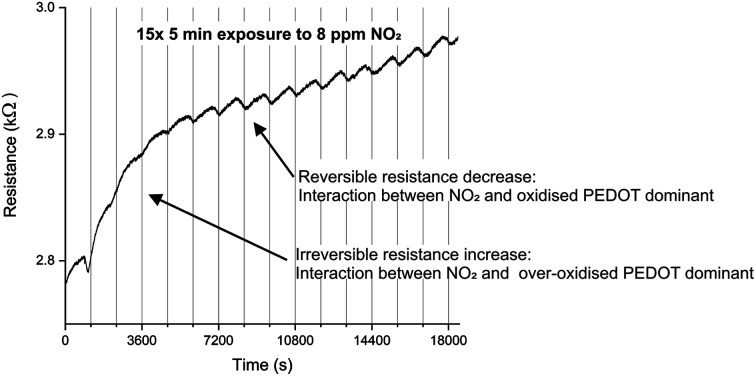

## Introduction

NO_2_ is a hazardous gas which is present in the atmosphere due to natural sources as well as combustion of fossil fuels and industrial activity. Accurate detection of NO_2_ is important for various safety applications. The World Health Organization 1 hour guideline of maximum exposure is 100 ppb and their annual average air quality guideline is 20 ppb.^1^

Hand-held NO_2_ detectors are commercially available at relatively low cost. Typically such hand-held devices operate in the 1–100 ppm range. This makes them suitable for applications in industry where they are used to signal imminent danger. However, for other applications and long term monitoring more sensitive sensing solutions with lower limits of detection are required.

To create highly sensitive small scale sensors, modern small scale materials hold significant potential. For example, one study reported highly sensitive NO_2_ sensors made using graphene,^2^ and another study made use of ZnO nanorods modified with SnO_2_ nanoparticles.^3^ Conducting polymers are an especially interesting class of nanomaterials for gas sensing because of the large range of materials available. Conducting polymers are relatively cheap and straightforward to process, and can be used in devices where the sensor operates at room temperature.

Conducting polymer based chemiresistive sensors using poly(3,4-ethylenedioxythiophene) (PEDOT) composites or nanomaterials for NO_2_ detection have been widely studied. Examples include PEDOT-PSS/TiO_2_ nanofibres,^4^ a WO_3_-PEDOT:PSS nanocomposite^5^ and a PEDOT–graphene composite.^6^ A study describing a chemiresistive sensor based on only PEDOT reports an irreversible sensor response. Although, as the authors suggest, their sensor can be used as a cumulative sensor, a reversible system would be preferable.^7^

Here we demonstrate sensitive and reversible sensing behaviour using only PEDOT as the sensor material. By adjusting the electrochemical polymerisation conditions, the relative contributions of oxidised and over-oxidised PEDOT to the sensing behaviour are controlled, and an optimised reversible sensor is created. Furthermore, we demonstrate a significantly improved sensitivity by using percolation networks of PEDOT instead of more traditional PEDOT thin films, as previously demonstrated for polypyrrole (PPy) percolation networks.^8,9^

## Materials and methods

Pt interdigitated electrodes (IDEs) consisting of 180 pairs of 5 μm wide electrodes with a 5 μm electrode separation on glass substrates (Micrux, Spain) were used after cleaning with concentrated nitric acid (90%) followed by sonication in ethanol (99.8%), methanol (99.9%), and acetone (99.8%). All solvents were purchased from Sigma-Aldrich (UK).

Poly-(3,4-ethylenedioxythiophene) (PEDOT) was grown on the IDEs using oxidative electrochemical polymerisation from a solution of 0.01 M 3,4-ethylenedioxythiophene (EDOT, Sigma-Aldrich) and 0.1 M lithium perchlorate (LiClO_4_, Sigma-Aldrich), in acetonitrile (99.8%, Sigma-Aldrich) using an Autolab PGSTAT204 potentiostat (Metrohom, Switzerland) and a PC equipped with Nova 11.1 software.^10^ The two connection pads of the IDEs were connected and together used as the working electrode. A Pt coil (BASi, USA) was used as the counter electrode and an Ag/AgCl (CH Instruments, USA) reference electrode was used. Using chronoamperometry, the potential between the working electrode and the reference electrode was kept at 1.3 V for 1–10 s.

After PEDOT growth, the samples were p-doped with perchlorate by placing them in a monomerless solution of 0.1 M LiClO_4_ in acetonitrile. The samples were p-doped for 60 s at 0.1–1.3 V. After doping, the sensors were rinsed with acetonitrile and left to dry in air. The same set-up with a monomerless solution was also used to obtain cyclic voltammograms of the PEDOT layers.

The sensors were tested in a custom made sensing chamber at atmospheric pressure and at room temperature. NO_2_ (10 ppm in N_2_) and N_2_ (for further dilution) cylinders were supplied by BOC (UK). The sensors were placed in the sensing chamber and left under N_2_ flow for 20 minutes to remove any impurities from the chamber or the sensing layer prior to the experiments. Next, 1–8 ppm NO_2_ was introduced into the chamber for 5 minutes. The concentrations were calculated from the relative flow rates of the two mass flow controllers (Alicat, USA) and the gas was mixed at a T-joint before entering the sensing chamber. A constant total flow rate of 500 sccm was maintained throughout the experiments using mass flow controllers. After exposure to NO_2_ the sensor was left under N_2_ flow for 15 minutes before the next exposure. Electrical connections from the sensor to a multimeter and a PC equipped with BenchVue software outside the sensing chamber allowed for continuous monitoring of the changes in the resistance of the PEDOT layer. During the sensing experiments a potential of 0.6 V was applied across the sensor electrodes and the current was measured, allowing the DC resistance to be determined.

The sensors were imaged using a Zeiss Merlin scanning electron microscope (SEM) at an accelerating voltage of 3 kV.

## Results and discussion

**Fig. 1:**
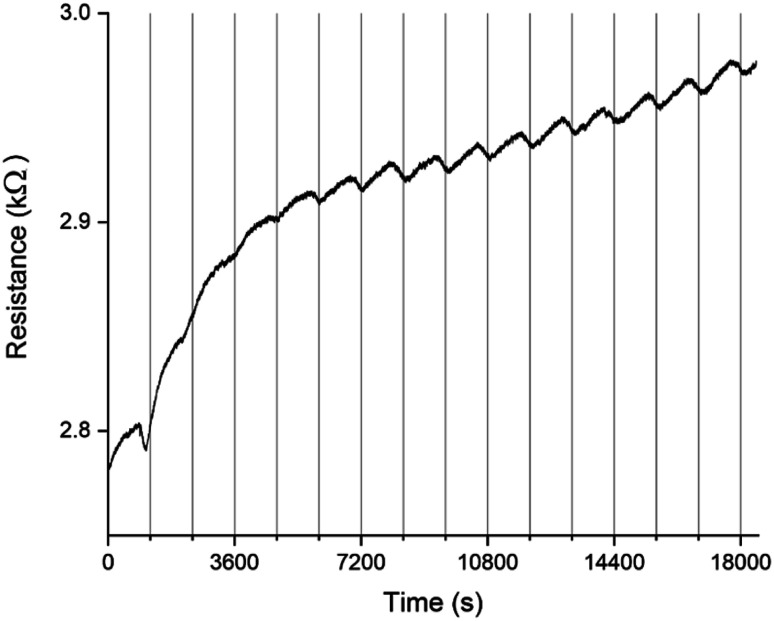
Typical sensing response of a sensor with a 2.8 kΩ starting resistance to 15 consecutive 5 minute long exposures of 8 ppm NO_2_, followed by 15 minute recovery periods under N_2_ between exposures. This sensing response is used to define the challenge that is solved in the rest of the paper. For the first 3 exposures an irreversible increase in resistance is seen, after which the behaviour changes so that reversible behaviour with good repeatability becomes dominant. The vertical lines are added as a guide to the eye, indicating the end of each NO_2_ exposure and the start of the recovery phase under N_2_ flow. Over the 5 hour timeframe of the experiment there is also a drift in the baseline resistance.

**Fig. 2:**
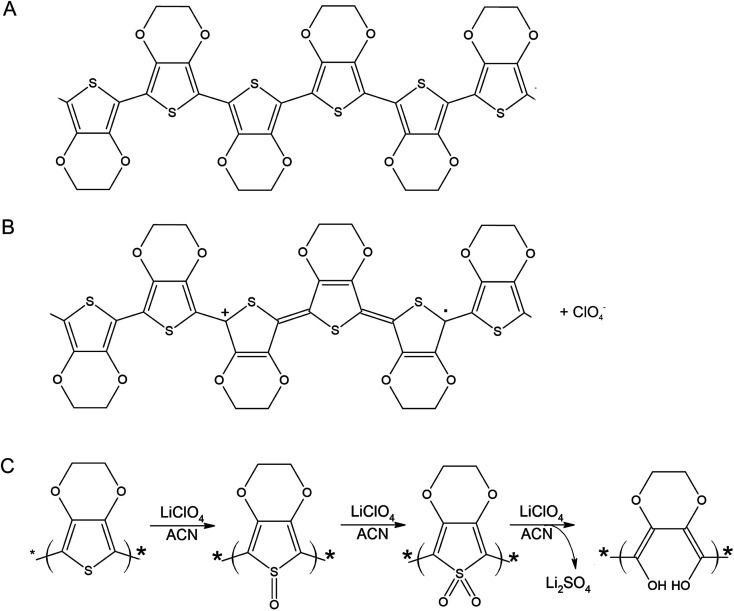
Molecular structure of PEDOT (A), oxidised PEDOT (B), and (C) a schematic of the proposed over-oxidation process of PEDOT with LiClO_4_ in acetonitrile (ACN) in 3 consecutive steps, adapted from Fan *et al.*^15^ An interaction of NO_2_ with oxidised PEDOT (B) is a reversible adsorption process, resulting in a reversible decrease in resistance. An interaction of NO_2_ with one of the stages of over-oxidised PEDOT (C) causes the PEDOT to move irreversibly to the next stage of over-oxidation, finally resulting in a disruption of conjugation and an irreversible increase in resistance.

A PEDOT based sensor was created by polymerisation at 1.3 V for 5 s, followed by doping at 1.3 V for 60 s, and had a starting resistance of 2.8 kΩ. The sensor was exposed 15 times to 8 ppm NO_2_ for 5 minutes, with a 15 minute recovery time under N_2_ flow between exposures (Fig. 1). The sensor response shown in Fig. 1 is used to define the challenge that will be resolved in the remainder of this paper. The first 3 exposures result in irreversible increases in resistance, but later exposures result in reversible decreases in resistance. These first 3 sensor responses are not what one might at first expect for the interaction of NO_2_ with PEDOT. One should expect to see a reversible decrease in resistance, rather than an irreversible increase. NO_2_ is a strong oxidiser and the PEDOT is p-doped.^11^ Therefore, the adsorption of NO_2_ should cause an increase in the majority charge carrier density of PEDOT, decreasing the resistance. This effect should be reversed when the NO_2_ desorbs from the PEDOT. However, our sensors and also those of other researchers^7^ do not show this behaviour. Instead, an irreversible increase in resistance is described in the literature and observed for the first 3 exposures in Fig. 1, which has been attributed to over-oxidation of PEDOT. Over-oxidation is a process in which the polymer is irreversibly damaged, causing an irreversible increase in resistance. It is thought that over-oxidation leads to a decrease in conjugation length of the delocalised π-electrons or a shortening of the polymer chains themselves.^12^ PEDOT films also undergo structural changes, such as increased crystallinity, upon over-oxidation.^13,14^ Because the electrochemical polymerisation potential of PEDOT is close to its over-oxidation potential, part of the polymer layer is over-oxidised during the polymerisation and doping processes. The interaction of the NO_2_ with the over-oxidised PEDOT causes a disruption in the conjugation of the polymer. Based on the literature, we propose a mechanism for the oxidation and eventual over-oxidation of PEDOT during the polymerisation and doping processes in the presence on LiClO_4_ (Fig. 2).^11,15,16^ This mechanism shows multiple over-oxidations steps. First oxygen binds to the sulphur atom in the thiophene ring. This is followed by the formation of lithium sulphate, eliminating the sulphur atom and opening the thiophene ring.

The sensor responses in Fig. 1 suggest that when a PEDOT film or percolation network is used as a chemiresistive gas sensor, even if the NO_2_ reacts with oxidised and over-oxidised PEDOT simultaneously, at first the reaction with the over-oxidised PEDOT dominates the sensor response. A reaction between over-oxidised PEDOT and NO_2_ causes the PEDOT to be over-oxidised even further following a similar mechanism as the over-oxidation in the presence of LiClO_4_ during electrochemical polymerisation (Fig. 2), eventually breaking the conjugation of the polymer chain. Therefore, both the preparation of the PEDOT layer and the exposure of the PEDOT layer to NO_2_ contribute to its over-oxidation. However, because the reaction between over-oxidised PEDOT and NO_2_ is an irreversible reaction, the number of potential reaction sites for this reaction decreases over time. On the other hand, the reaction with the oxidised PEDOT is reversible and, while there is a dynamic equilibrium of NO_2_ adsorbing and desorbing, the total number of potential reaction sites remains the same over time. This means that as time progresses the fraction of the available NO_2_ molecules that reacts with oxidised PEDOT instead of over-oxidised PEDOT increases and the reversible decrease in resistance becomes the dominant sensor response. This shift towards the reversible response also suggests that NO_2_ is not a strong enough oxidiser to convert the oxidised PEDOT into over-oxidised PEDOT. The example in Fig. 1 demonstrates that once the over-oxidised PEDOT has reacted with NO_2_ a stable and reversible sensor with good repeatability is obtained.

Because for sensing applications reversible behaviour is preferred, some research has been directed towards finding ways around the issues associated with over-oxidation. For example the use of composites^17^ or control over the pH of the system^18^ is used to protect the PEDOT from over-oxidation. The remainder of this paper is focused on controlling the balance between oxidised and over-oxidised PEDOT through changes to the sensor production processing steps, making it possible to access reversible and irreversible sensor behaviour in a controlled manner and without the use of additional materials.

There are several factors that can contribute to oxidation and over-oxidation of a conductive polymer layer. The electrochemical conditions of both the electrochemical polymerisation and doping processes will be our focus. It is known that polymerisation, p-doping, and over-oxidation can all occur at the same time and that there is significant overlap between the potential range for over-oxidation and the potential range for reversible oxidation and reduction of PEDOT.^19,20^ It is also known that exposure to light and oxygen in ambient conditions can, by causing over-oxidation, contribute to the aging of the polymer material.^21^ In this study the aging effects are minimized by using freshly prepared PEDOT sensors at all times.

### PEDOT layer thickness and (over-)oxidation

**Fig. 3:**
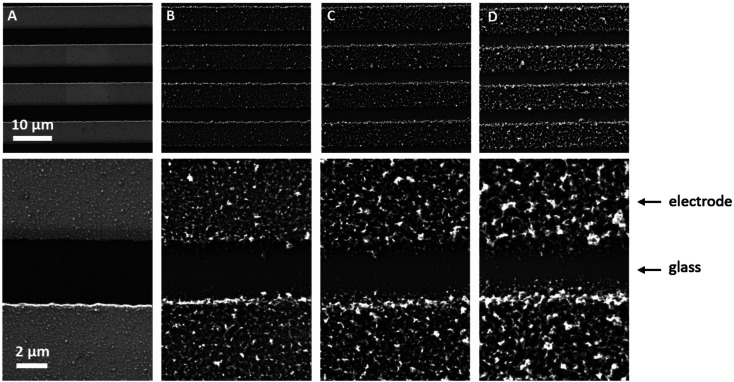
SEM images of PEDOT electrochemically deposited on glass substrates with Pt interdigitated electrodes with a 5 μm separation for (A) 2 s, (B) 5 s, (C) 7 s, and (D) 10 s. As expected, longer deposition times result in more PEDOT being deposited. Initially most of the PEDOT grows on the Pt electrodes. As the deposition time is increased both the amount of PEDOT on the electrodes and between the electrodes increases. The top row shows four Pt electrodes, and the bottom row shows higher magnification images showing two electrodes with the glass substrate in the centre.

**Fig. 4:**
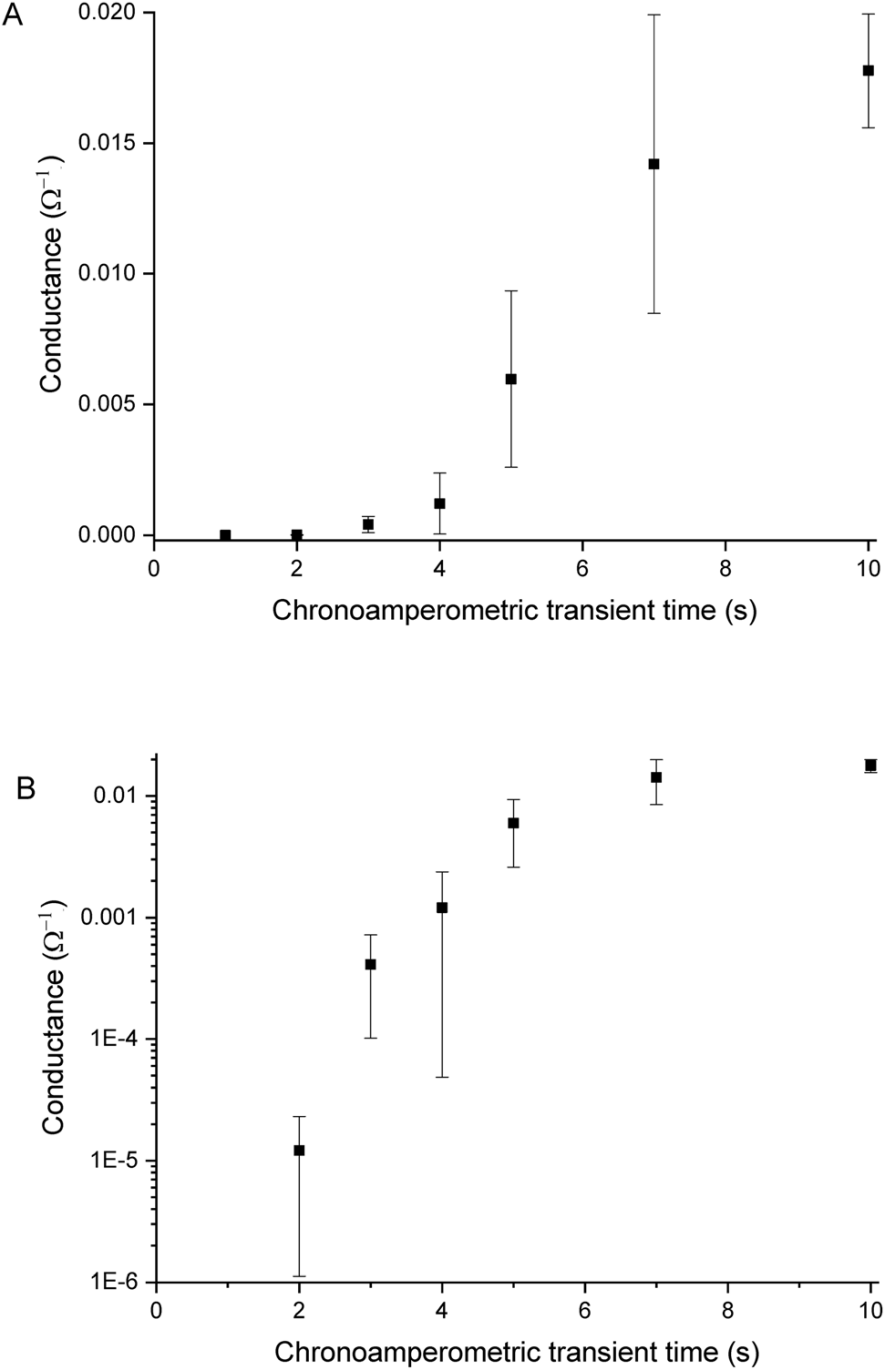
Conductance *vs.* chronoamperometric deposition time for PEDOT on glass substrates with Pt interdigitated electrodes with a 5 μm separation (A). The conductance increases with increasing deposition time. Plotting the same data in a semi-logarithmic plot highlights the large change in conductance caused by depositing slightly more polymer in the percolation region between 2 and 7 s (B). The error bars represent the standard deviation of 3 independent experiments.

Sensors with various PEDOT coverages were grown on the Pt IDEs using chronoamperometry for 1–10 s. All of these sensors were doped at 1.3 V for 60 s in a monomerless solution. As expected, SEM images show that the longer chronoamperometric transient times resulted in more PEDOT being grown on the sample (Fig. 3). Initially the polymer grows on the Pt electrodes. Then, for longer transient times, more polymer is grown on the electrodes and polymer starts to grow on the insulating glass substrate between the electrodes. Eventually the gaps between the electrodes are bridged and a PEDOT electrical percolation network is formed. Even longer transient times result in PEDOT thin films. This is confirmed by the electrical resistance measured between the two sides of the IDEs. For short transient times there is no electrical connection between the electrodes. For longer transient times connections are formed between the electrodes and the conductance increases. In the phase where there is sharp increase in conductance, the polymer layer acts as a percolation network. For longer transient times the conductance levels off as the polymer layer becomes thin-film-like. This is shown in the percolation data in Fig. 4 where the electrical conductance between the IDEs is plotted against the chronoamperometric transient time, which is equivalent to plotting conductance against amount of polymer deposited. When comparing the information in Fig. 3 and 4 it is interesting to note that the SEM images from 5 s PEDOT growth (Fig. 3b) do not appear to show polymer bridging, however the electrical conduction measurements (Fig. 4) demonstrate that bridging has occurred. This shows that SEM imaging is limited in being able to show the full extent of PEDOT growth. Previous work on PPy based NH_3_ sensors has shown that sensors operating in the steep part of the percolation curve are significantly more sensitive than those in the thin film regime. This is because near the percolation threshold small local changes in the resistance of the polymer connections due to analyte adsorption have a much bigger impact on the resistance of the network as a whole than in the case of a thin film.^8^ The large effect on the conductance caused by small variations in the networks is also why, in the percolation region, there is a relatively large variation in conductance for networks grown under the same conditions and for the same chronoamperometric transient time, resulting in the relatively large error bars in Fig. 4.

**Fig. 5:**
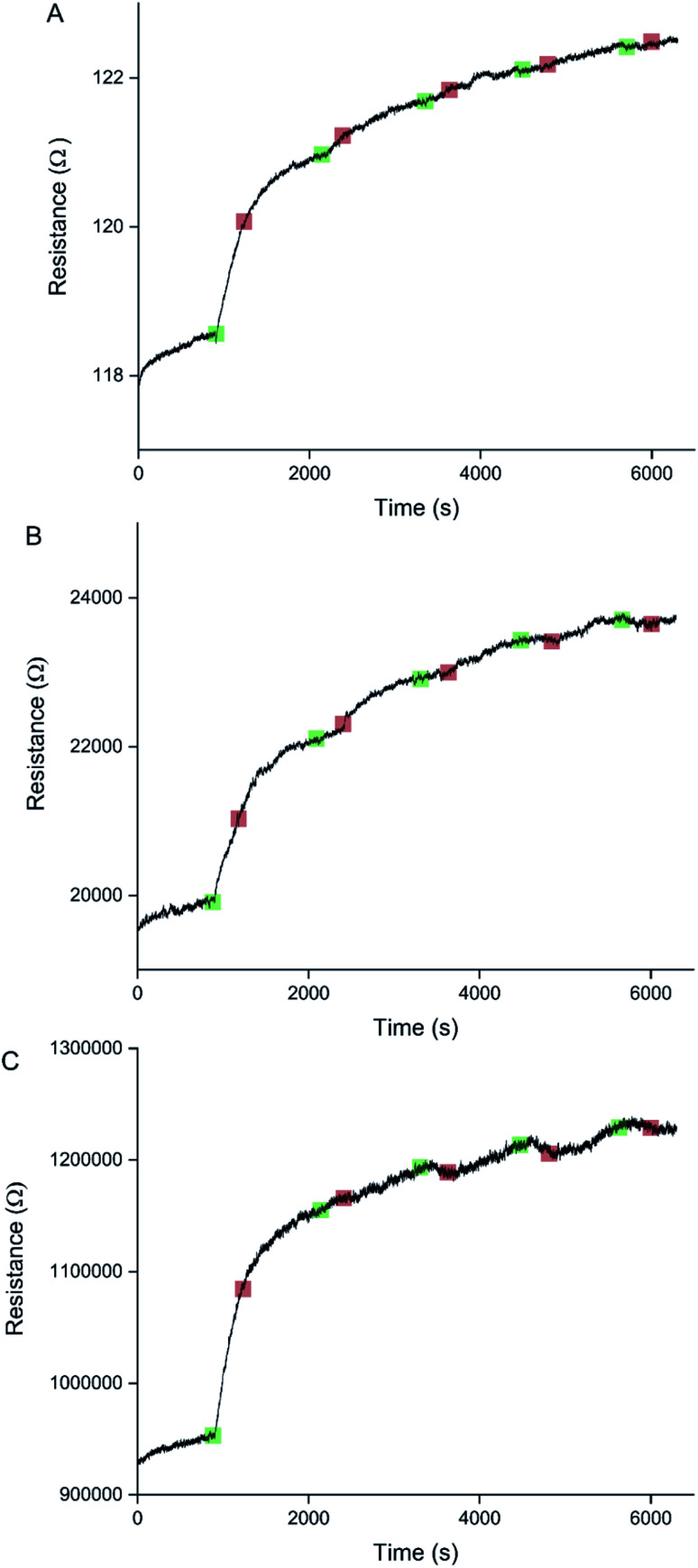
Raw sensor responses of a thin-film-like sensor (A), an intermediate sensor (B), and a sensor close to the percolation threshold (C). All three sensors were exposed 4 times to 8 ppm NO_2_ for 5 minutes, with a 15 minute recovery time under N_2_ flow between exposures. Green indicates the start of an NO_2_ exposure and red indicates the start of the recovery phase under N_2_ flow. The sensor closest to the percolation threshold (C) has the highest sensitivity, defined as Δ*R*/*R*_0_ × 100%. Although for all 3 sensors the response to the first exposure to NO_2_ results in an irreversible increase in resistance, the sensor responses are not consistent.

Sensors at different points along the percolation curve, from close to the percolation threshold to thin-film-like sensors, were placed under N_2_ flow in the sensor testing chamber for 20 minutes. Next the sensors were exposed 4 times to 8 ppm NO_2_, for 5 minutes per exposure and with a 15 minute recovery time under N_2_ flow between exposures (Fig. 5). A comparison of the sensor responses of these sensors shows that PEDOT sensors closer to the percolation threshold have a higher sensitivity than thin film sensors. For example, for the first 8 ppm exposure for the sensors in Fig. 3 we observed sensitivities, expressed as Δ*R*/*R*_0_ × 100%, of 0.79%, 5.77%, and 12.9% for sensors with starting resistances of 120 Ω, 20 kΩ, and 950 kΩ, or chronoamperometric transient times of 10 s, 4 s, and 2 s, respectively. This observation, that the sensitivity is greatest for PEDOT polymer percolation networks that are operated in the steepest part of the percolation response curve, is in agreement with previous work on PPy percolation network sensors.^8^

The first exposure of each of the sensors in Fig. 5 results in an irreversible increase in resistance, in line with the response of PEDOT films to NO_2_ described in the literature.^7^ However, none of these sensors have a consistent sensor response for all 4 consecutive exposures to NO_2_. For the thin-film-like sensor (A) the second, third, and fourth exposures result in irreversible increases in resistance with smaller magnitudes than for the first exposure. For the sensor closest to the percolation threshold (C) later exposures result in a small reversible decrease in resistance. Sensor B is an intermediate case, displaying some smaller reversible decreases in resistance at a later onset than for sensor C.

**Fig. 6:**
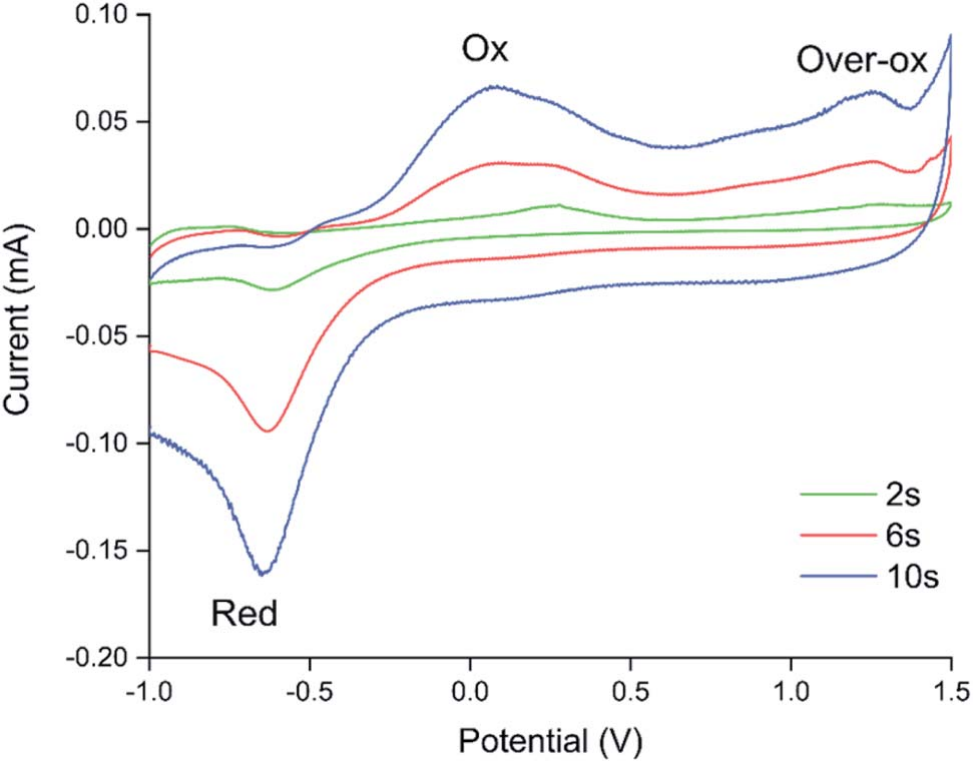
Cyclic voltammograms for 3 sensors at different points along the percolation curve, created by chronoamperometric deposition for 2 s, 6 s, and 10 s at 1.3 V, followed by doping at 1.3 V for 60 s. As expected longer deposition times result in larger oxidation (0.1–0.3 V), over-oxidation (1.2–1.3 V) and reduction (−0.6 V) peaks. The relative magnitudes of the oxidation and over-oxidation peaks are similar for all three sensors.

Because electrochemical polymerisation, p-doping, and over-oxidation can all occur at the same time, the PEDOT is already being over-oxidised during the electrochemical polymerisation process. The resulting polymer layer consists of oxidised polymer and polymer in the various over-oxidation stages (Fig. 2). This explains why in Fig. 5 sensor A, with a 10 s deposition time, only displays an irreversible response, whereas sensor B, with a deposition time of 5 s, displays a mixture of irreversible and reversible responses, and sensor C, with a 2 s deposition, has the most pronounced reversible response of the three sensors. The longer the deposition time, the more over-oxidised PEDOT is present. This is supported by cyclic voltammograms of sensors deposited for 2 s, 6 s, and 10 s at 1.3 V, followed by doping at 1.3 V for 60 s (Fig. 6). In cyclic voltammograms over-oxidation is characterized by a second oxidation peak. In agreement with the literature, this second oxidation peak does not have a corresponding reduction peak in the reverse sweep.^22,23^ The lack of corresponding reduction peak further highlights the irreversible nature of the over-oxidation process. Fig. 6 shows that, as expected longer polymerisation times result in larger oxidation (0.1–0.3 V), over-oxidation (1.2–1.3 V) and reduction (−0.6 V) peaks. The relative magnitudes of the oxidation and over-oxidation peaks are similar for all three samples. Because initially the net sensor response is dominated by the reaction with over-oxidised PEDOT, this means that the irreversible response becomes more dominant at longer deposition times. This also explains why only the irreversible response has been previously reported in the literature. Traditionally chemiresistive sensors consist of a polymer thin film, at relatively long deposition times, and are thus dominated by the irreversible response due to over-oxidation.

### Doping potential and (over-)oxidation

It is clear that the duration for which a potential is applied has a large effect on the oxidation state of the resulting PEDOT layer. The polymerisation potential itself cannot be decreased to move away from the potential range at which over-oxidation takes place, because at a lower potential polymerisation does not occur. However, the doping potential can be changed to alter the properties of the resulting polymer layer.

To study the effect of the doping potential, 8 sensors were given different doping treatments and their sensing responses were compared. Here, PEDOT thin film sensors were used because they are easier to reproduce than sensors close to the percolation threshold. Sensors close to the percolation threshold display a large amount of variation in their starting resistances, even for sensors created using the same preparation procedures, because close to the percolation threshold small changes to the polymer network have a relatively large impact on the resistance of the network as a whole. Furthermore, using thin-film-like sensors enables better comparison to existing literature, which is mostly on thin-film based devices.

**Fig. 7:**
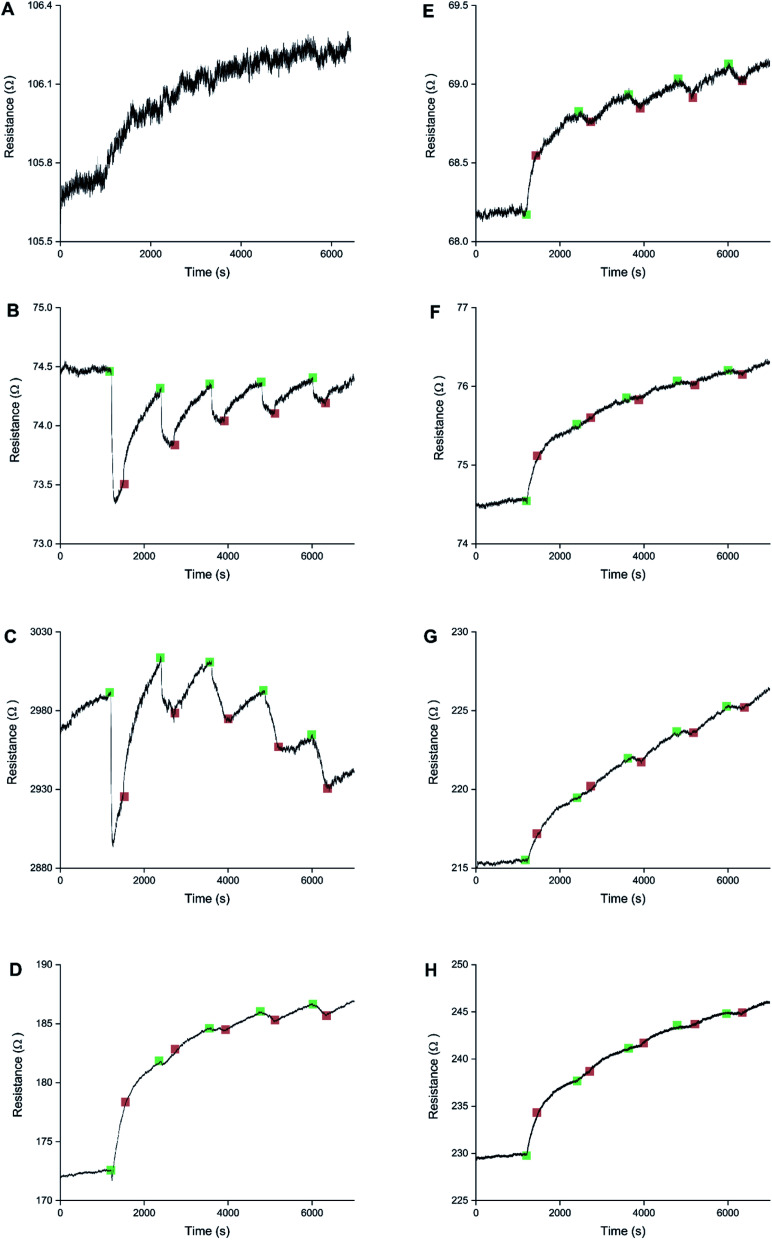
Sensing responses to 5 exposures of 8 ppm NO_2_ for 8 PEDOT thin film sensors, all created by chronoamperometric deposition at 1.3 V for 10 s, after different doping treatment: no doping (A), and doping for 60 s at 0.1, 0.3, 0.5, 0.7, 0.9, 1.1, and 1.3 V, respectively (B–H). The sensor (A) that wasn't doped has a very poor signal-to-noise ratio. The other sensors show a clear trend of reversible responses for low doping potentials (B and C) and irreversible responses for high doping potentials (F–H). Intermediate doping potentials (D and E) result in a crossover situation where the first response(s) is/are irreversible and later responses are reversible. Variations in the starting resistance, despite identical sensor preparation, are inherent to chronoamperometric polymerisation of thin polymer layers and can be decreased by increasing the layer thickness.

The sensors were created by depositing PEDOT for 10 s at 1.3 V. Next the sensors were either not doped, or doped at 0.1, 0.3, 0.5, 0.7, 0.9, 1.1, or 1.3 V for 60 s (Fig. 7). All 8 sensors underwent 5 cycles of exposure to 8 ppm NO_2_ for 5 minutes and recovery under N_2_ for 15 minutes. The un-doped sensor had a very poor signal-to-noise ratio. For the other sensors a clear trend is visible. Low doping potentials result in sensors that have reversible decreases in resistance when exposed to NO_2_, caused by the oxidised PEDOT. High doping potentials result in irreversible increases in resistance, like previously reported in the literature, caused by the over-oxidised PEDOT. Intermediate doping potentials display crossover behaviour. For intermediate doping potentials the first response or responses are dominated by the irreversible over-oxidised behaviour, while later responses display reversible oxidised behaviour. Sometimes the 2 behaviours can be seen to be competing within the same exposure cycle.

### Designing a reversible sensor

This means that there are 2 ways to control the balance between oxidised and over-oxidised PEDOT, and therefore the (ir)reversible sensing behaviour: the chronoamperometric transient time during polymerisation and the doping potential. Sparser networks, closer to the percolation threshold, and low doping potentials both result in a reversible decrease in resistance, whereas thicker films and higher doping potentials result in irreversible increases in resistance.

**Fig. 8:**
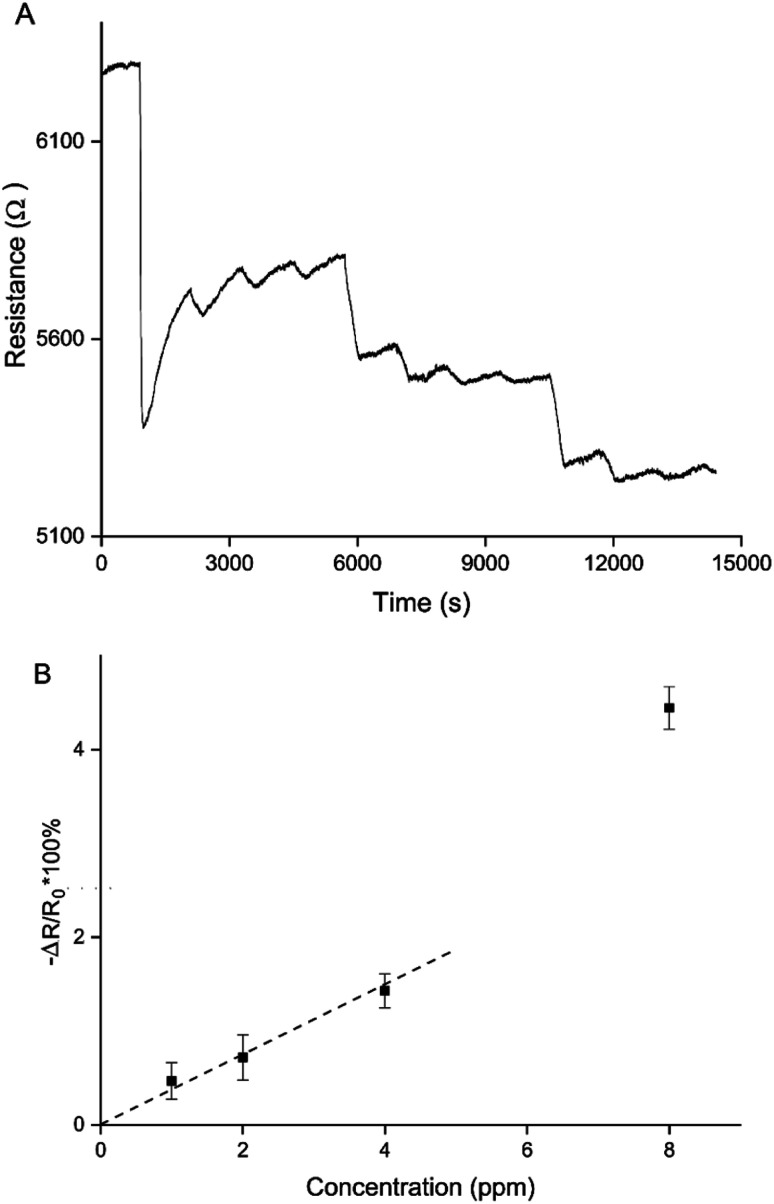
Sensor responses to 3 rounds of 8 ppm, 4 ppm, 2 ppm, and 1 ppm NO_2_ of a PEDOT percolation network based sensor optimised to give a reversible sensor response (A). Average sensor responses in Δ*R*/*R*_0_ × 100% for 3 exposures per concentration for the same sensor (B). The data for 8 ppm was not included in the trend line because the data for 8 ppm is a clear outlier, resulting in a larger change in sensor response than expected.

Making use of both of these control mechanisms, a sensitive and reversible NO_2_ sensor using only PEDOT was created (Fig. 8). A PEDOT percolation network sensor was created by chronoamperometric polymerisation at 1.3 V for 5 s and doping at 0.1 V for 60 s. This sensor was exposed 3 times to 8 ppm, 4 ppm, 2 ppm and 1 ppm NO_2_. A sensitive and reversible sensor was created by controlling the PEDOT layer thickness, or polymerisation time, and doping potential (Fig. 8A). Fig. 8B shows the average responses for the 3 exposures per NO_2_ concentration for this sensor. Using this calibration curve a limit of detection (LOD) can be calculated. The LOD is defined as the gradient of the trend line of the calibration curve divided by 3 times the standard deviation of the baseline noise level before the first exposure. Because the response to 8 ppm was an outlier, resulting in a significantly larger resistance change than expected, it was not included in the trend line. Based on the trend line, the LOD of detection for this particular sensor is 907 ± 102 ppb. This matches the most sensitive commercially available handheld NO_2_ detectors.

## Conclusions

In conclusion, by controlling the polymer coverage and the doping conditions sensitive and reversible PEDOT based chemiresistive NO_2_ sensors were developed. By operating PEDOT sensors close to the percolation threshold higher sensitivities can be achieved compared to more traditional thin film based sensors. Furthermore, by controlling the polymer coverage and doping potential the oxidised regime can be reliably accessed, resulting in reversible sensors.

Although this is an important step towards creating reversible NO_2_ sensors based on only PEDOT, more work on the effects of for example humidity and other competing vapours in the environment has to be done prior to practical implementation of these sensors.

Finally, it is known that PPy^24^ and polyindole (PIn)^25^ can also be over-oxidised, and a similar effect of oxidation and over-oxidation on the sensor response was also observed for PPy sensors responding to NO_2_ in preliminary studies, but this requires further work.

## Conflicts of interest

There are no conflicts to declare.

## Supplementary Material
